# Predictors of disease progression in HIV infection: a review

**DOI:** 10.1186/1742-6405-4-11

**Published:** 2007-05-14

**Authors:** Simone E Langford, Jintanat Ananworanich, David A Cooper

**Affiliations:** 1 Monash University, Melbourne, Australia; 2 The HIV Netherlands Australia Thailand Research Collaboration, Bangkok, Thailand; 3 The National Centre in HIV Epidemiology and Clinical Research, Sydney, Australia, University of New South Wales, Sydney, Australia

## Abstract

During the extended clinically latent period associated with Human Immunodeficiency Virus (HIV) infection the virus itself is far from latent. This phase of infection generally comes to an end with the development of symptomatic illness. Understanding the factors affecting disease progression can aid treatment commencement and therapeutic monitoring decisions. An example of this is the clear utility of CD4+ T-cell count and HIV-RNA for disease stage and progression assessment.

Elements of the immune response such as the diversity of HIV-specific cytotoxic lymphocyte responses and cell-surface CD38 expression correlate significantly with the control of viral replication. However, the relationship between soluble markers of immune activation and disease progression remains inconclusive. In patients on treatment, sustained virological rebound to >10 000 copies/mL is associated with poor clinical outcome. However, the same is not true of transient elevations of HIV RNA (blips). Another virological factor, drug resistance, is becoming a growing problem around the globe and monitoring must play a part in the surveillance and control of the epidemic worldwide. The links between chemokine receptor tropism and rate of disease progression remain uncertain and the clinical utility of monitoring viral strain is yet to be determined. The large number of confounding factors has made investigation of the roles of race and viral subtype difficult, and further research is needed to elucidate their significance.

Host factors such as age, HLA and CYP polymorphisms and psychosocial factors remain important, though often unalterable, predictors of disease progression. Although gender and mode of transmission have a lesser role in disease progression, they may impact other markers such as viral load. Finally, readily measurable markers of disease such as total lymphocyte count, haemoglobin, body mass index and delayed type hypersensitivity may come into favour as ART becomes increasingly available in resource-limited parts of the world. The influence of these, and other factors, on the clinical progression of HIV infection are reviewed in detail, both preceding and following treatment initiation.

## Review

Throughout the clinically latent period associated with Human Immunodeficiency Virus (HIV) infection the virus continues to actively replicate, usually resulting in symptomatic illness [1-3]. Highly variable disease progression rates between individuals are well-recognised, with progression categorised as rapid, typical or intermediate and late or long-term non-progression [1,4]. The majority of infected individuals (70–80%) experience intermediate disease progression in which they have HIV-RNA rise, CD4+ T-cell decline and development of AIDS-related illnesses within 6–10 years of acquiring HIV. Ten to 15% are rapid progressors who have a fast CD4+ T-cell decline and occurrence of AIDS-related events within a few years after infection. The late progressors (5%), can remain healthy without significant changes in CD4 count or HIV-RNA for over 10 years [4].

**Figure 1:**
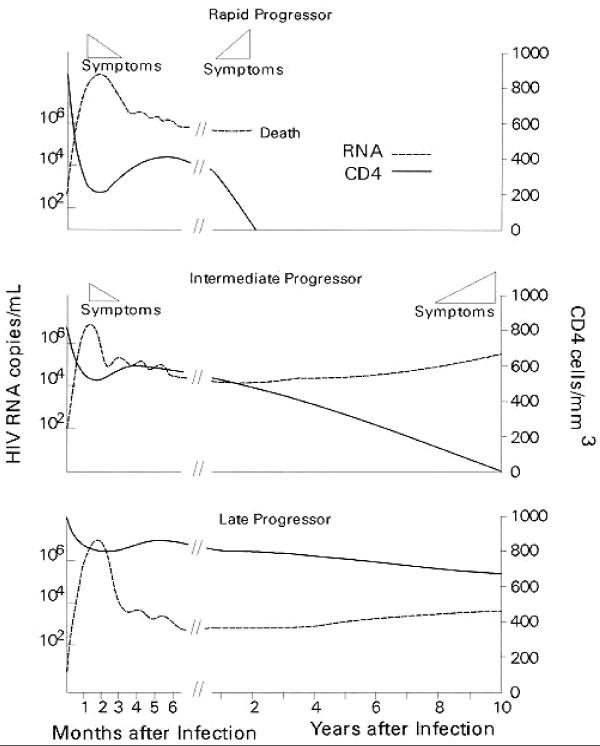
General pattern of the natural history of HIV-RNA levels and CD4 counts at three rates of disease progression [5] (Reproduced from Figure 1, HIV InSite Knowledge Base, with permission).

While Figure 1[5] demonstrates the existence of a relationship between high plasma HIV-RNA, low peripheral CD4+ T-cell count and rapidity of disease progression, many of the determinants of this variation in progression are only partially understood. Knowledge of prognostic determinants is important to guide patient management and treatment. Much research has focussed on many different facets of HIV pathogenesis and possible predictive factors, covering immunological, virological and host genetic aspects of disease. Current therapeutic guidelines take many of these into account but their individual significance warrants review [6].

## Immunological factors

### T-cell count and function

#### CD4+ T-cells

CD4+ T-cells are fundamental to the development of specific immune responses to infection, particularly intracellular pathogens. As the primary target of HIV, their depletion severely limits the host response capacity. HIV largely infects activated cells, causing the activated T-cells directed against the virus to be at greatest risk of infection [7]. The ability of the immune system to mount a specific response against HIV is a key factor in the subsequent disease course [8]. Long-term non-progressors appear to have better lymphoproliferative responses to HIV-specific antigens than those with more rapid progression [8].

The CD4+ T-cell count is the most significant predictor of disease progression and survival [9-15], and the US Department of Health and Human Services (DHHS) ART treatment guidelines recommends treatment commencement be based on CD4+ T-cell count in preference to any other single marker [6]. Table 1 shows the results of the CASCADE collaboration^i ^(see Appendix 1 for details) analysis of an international cohort of 3226 ART-naïve individuals with estimable dates of seroconversion. Each CD4 count was considered to hold predictive value for no more than the subsequent 6 month period, with individual patients contributing multiple 6 month periods of follow up [10]. Lower CD4 counts are associated with greater risk of disease progression. CD4 counts from 350–500 cells/mm^3 ^are associated with risks of ≤5% across all age and HIV-RNA strata, while the risk of progression to AIDS increases substantially at CD4 counts <350 cells/mm^3^, the greatest risk increase occurring as CD4 counts fall below 200 cells/mm^3^. The risk of disease progression at 200 cells/mm^3^, the threshold for ART initiation in resource-limited settings, is generally double the risk at 350 cells/mm^3^, the treatment threshold in resource-rich countries [10].

**Table 1:** Predicted 6 month risk of AIDS according to age, current CD4+ cell count and viral load, based on a Poisson regression model

**Viral load (copies/mL)**	**Predicted risk (%) at current CD4 count (× 10^6 ^cells/L)**									
**Age**	**50**	**100**	**150**	**200**	**250**	**300**	**350**	**400**	**450**	**500**

**25 years**										
3000	*6.8*	*3.7*	*2.3*	1.6	1.1	0.8	0.6	0.5	0.4	0.3
10 000	*9.6*	*5.3*	*3.4*	*2.3*	1.6	1.2	0.9	0.7	0.5	0.4
30 000	**13.3**	*7.4*	*4.7*	*3.2*	*2.2*	1.6	1.2	0.9	0.7	0.6
100 000	**18.6**	**10.6**	*6.7*	*4.6*	*3.2*	*2.4*	1.8	1.4	1.1	0.8
300 000	***25.1***	**14.5**	*9.3*	*6.3*	*4.5*	*3.3*	*2.5*	1.9	1.5	1.2
										
**35 years**										
3000	*8.5*	*4.7*	*3.0*	*2.0*	1.4	1.0	0.8	0.6	0.5	0.4
10 000	**12.1**	*6.7*	*4.3*	*2.9*	*2.0*	1.5	1.1	0.9	0.7	0.5
30 000	**16.6**	*9.3*	*5.9*	*4.0*	*2.8*	*2.1*	1.6	1.2	0.9	0.7
100 000	***23.1***	**13.2**	*8.5*	*5.8*	*4.1*	*3.0*	*2.3*	1.7	1.3	1.1
300 000	***30.8***	**18.0**	**11.7**	*8.0*	*5.7*	*4.2*	*3.1*	*2.4*	1.9	1.5
										
**45 years**										
3000	**10.7**	*5.9*	*3.7*	*2.5*	1.8	1.3	1.0	0.7	0.6	0.5
10 000	**15.1**	*8.5*	*5.4*	*3.6*	*2.6*	1.9	1.4	1.1	0.8	0.7
30 000	***20.6***	**11.7**	*7.5*	*5.1*	*3.6*	*2.6*	*2.0*	1.5	1.2	0.9
100 000	***28.4***	**16.5**	**10.6**	*7.3*	*5.2*	*3.8*	*2.9*	*2.2*	1.7	1.3
300 000	***37.4***	***22.4***	**14.6**	**10.1**	*7.2*	*5.3*	*4.0*	*3.1*	*2.4*	1.9
										
**55 years**										
3000	**13.4**	*7.5*	*4.7*	*3.2*	*2.3*	1.7	1.2	0.9	0.7	0.6
10 000	**18.8**	**10.7**	*6.8*	*4.6*	*3.3*	*2.4*	1.8	1.4	1.1	0.8
30 000	***25.4***	**14.6**	*9.4*	*6.4*	*4.6*	*3.3*	*2.5*	1.9	1.5	1.2
100 000	***34.6***	***20.5***	**13.3**	9.2	6.5	4.8	3.6	2.8	2.2	1.7
300 000	***44.8***	***27.5***	**18.2**	**12.6**	*9.1*	*6.7*	*5.0*	*3.9*	*3.0*	*2.4*

<2%, *risk 2–9.9%*, **risk 10–19.9%, *risk ≥20%***

This table is reproduced from Table 4 in [10]

**Figure 2:**
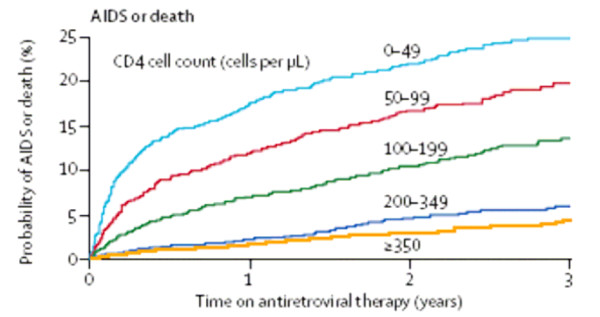
Kaplan Meier plots of the probability of progression to AIDS or death according to baseline CD4 count [20] (reproduced with permission).

Use of the CD4 count as a means of monitoring ART efficacy is well established [6,16]. In particular, measurement of the early response in the first six months of therapy has strong predictive value for future immunological progression [17,18]. Baseline CD4 count is predictive of virological failure, Van Leth et al. [19] finding a statistically significant correlation between a baseline CD4 count of <200 cell/mm^3 ^and HIV-RNA >50 copies/mL at week 48 of therapy. Figure 2 shows the importance of baseline CD4 count as a predictor of disease progression; each stratum of CD4 count <200 cell/mm^3 ^at time of HAART initiation being associated with an increasingly worse prognosis [20]. Immunological recovery is largely dependent on baseline CD4 count and thus the timing of ART initiation is important in order to maximise the CD4+ T-cell response to therapy [20].

It is important to note that within-patient variability in CD4+ T-cell quantification can occur and so care must be taken to ensure measurements are consistently performed by the same method for each patient [9].

#### CD8 T-lymphocyte function

The influence of CD8+ T-lymphocyte function on HIV disease progression is of considerable interest as cytotoxic T-lymphocytes (CTLs) are the main effector cells of the specific cellular immune response. Activated by CD4+ T-helper cells, anti-HIV specific CD8+ T-cells have a crucial role to play in the control of viremia [21], increasing in response to ongoing viral replication [22]. Further, the diversity of HIV-specific CTL responses correlates with the control of viral replication and CD4 count, indicating the need for a response to a broad range of antigens to achieve a maximum effect [23,24]. Low absolute numbers of HIV-specific CD8+ T-cells correlate with poor survival outcomes in both ART-naïve and experienced patients, providing additional evidence for the significance of the CTL response [23,25,26].

### Immune activation

Chronic immune activation is a characteristic of HIV disease progression. Immune-activation-driven apoptosis of CD4+ cells, more than a direct virological pathogenic effect, is responsible for the decline in CD4+ T-lymphocytes seen in HIV infection [27]. HIV triggers polyclonal B cell activation, increased T cell turnover, production of proinflammatory cytokines and increased numbers of activated T cells [28]. CD4+ T cells that express activation markers such as CD69, CD25, and MHC class II are a prime target for HIV infection and a source of active HIV replication. Increased numbers of these activated T cells correlate with HIV disease progression [29-31]. Another important surface marker of cell activation is CD38. In HIV negative individuals, CD38 is expressed in relatively greater numbers by naïve lymphocytes, while in HIV infection, memory T-cells, particularly CD8+ memory T-cells, express the largest quantities of CD38 [32,33]. CD8+CD38+T-cell levels correlate strongly with HIV-RNA levels, decreasing with ART-induced virological suppression and increasing with transient viremia, suggesting that continuously high levels of CD38+ cells may be an indicator of ongoing viral replication [32-35]. Indeed, HIV replication has been nominated the main driving force behind CD8+ T-cell activation [32,33]. Similar to the stabilization of HIV-RNA levels following initial infection, an immune activation "set point" has also been described and shown to have prognostic value [36].

Despite the strength of the relationship with HIV-RNA, the search for a clear association between CD8+ T-cell activation and CD4 count has resulted in conflicting findings [32,33,35,37]. In contrast, CD4+ T-cell activation has a considerable influence on CD4+ T-cell decline [27,34,35]. One prospective study of 102 seroconverters has found that CD8+CD38+ proportions lose their prognostic significance over time and only elevated CD4+CD38+ percentage is associated with clinical deterioration at 5 years follow up [27]. The clinical value of monitoring CD38 expression is yet to be clarified, however, there is no doubt that disease progression is related to both CD4+ and CD8+ T-cell activation as indicated by expression of CD38.

A switch from T-Helper Type 1 (TH1) to T-Helper Type 2 (TH2) cytokine response is seen in HIV-related immune dysfunction and is associated with HIV disease progression. TH1 cytokines such as interleukin (IL)-2, IL-12, IL-18 and interferon-γ promote strong cellular responses and early HIV viremic control while TH2 cytokines, predominantly modulated by IL-1β, IL-4, IL-6, IL-10 and tumor necrosis factor-α (TNF-α), promote HIV viral replication and dampen cellular response to HIV [8,38]. HIV-positive individuals have high plasma IL-10 levels, reduced production of IL-12 and poor proliferation of IL-2 producing CD4+ central memory T-cells [30,39,40]. Levels of pro-inflammatory cytokines such as TNF-α are also increased, causing CD8+ T-cell apoptosis [1,40].

Soluble markers of immunological activity have been the focus of many studies over the years in the hope that they will show utility as prognostic indicators. Unfortunately, the product of these endeavours is a large number of studies with apparently conflicting results, some studies linking elevated levels of these markers with more rapid disease progression [41-46], and others finding no correlation [46-48]. Factors investigated include neopterin [41-43,45,46,48], β_2_-microglobulin [42,46-48], tumour necrosis factor type II receptor [41,46], tumour necrosis factor receptor 75 [45], endogenous interferon [43] and tumour necrosis factor-α [25]. The lack of specificity of these markers for HIV infection appears to curtail their utility. Current treatment guidelines make no mention of their use for either disease or therapeutic monitoring [6,49,50]. As the immune response to HIV is clarified with further research, the utility of monitoring these immune modulators may become more apparent.

## Virological factors

### HIV-RNA

The value of HIV-RNA quantification as a prognostic marker has long been established [6,51,52]. An approximately inverse relationship to the CD4+ T-cell count and survival time has been observed in around 80% of patients [53,54]. Higher HIV-RNA levels are associated with more rapid decline of CD4+ T-cells, assisting prediction of the rate of CD4 count decline and disease progression. However, once the CD4 count is very low (<50–100 cells/mm^3^), the disease progression risk is so great that HIV-RNA levels add little prognostic information [25,54-56]. The correlation between CD4 count and disease progression seen clearly in Table 1 has already been described [10]. Further highlighting the risk of AIDS in those with CD4 counts of 200–350 cell/mm^3 ^(the current threshold for ART initiation), a four-fold risk increase can be seen between those with a HIV-RNA of 3000 copies/mL and those with ≥300 000 copies/mL, even within the same age bracket. Additionally, there is a considerable increase in risk of disease progression in those with HIV-RNA >100 000 copies/mL across all age and CD4 strata.

Higher baseline HIV-RNA levels in early infection have been associated with faster CD4+ T-cell decline over the first two years of infection [15,57]. Research has suggested that HIV-RNA levels at later time points are better indicators of long term disease progression than levels at seroconversion, with the viral load reaching a stable mean or 'set point' around one year after infection [4,12,52,58,59]. Indicative of the efficiency of immunological control of viral replication, this set point is strongly associated with the rate of disease progression as can be inferred from Figure 1[1,52,59-62]. Desquilbet et al. [63] studied the effect of early ART on the virological set point by starting treatment during Primary HIV Infection (PHI) and then ceasing it soon after. They found the virological set point was mainly determined by pre-treatment viral load, early treatment having minimal reducing effect.

Treatment response has been strongly linked to the baseline HIV-RNA level; Van Leth et al. [19] finding that patients with a HIV-RNA >100 000 copies/mL were almost 1.5 times more likely to experience virological failure (HIV-RNA >50 copies/mL) after 48 weeks of treatment than those with HIV-RNA <100 000 copies/mL.

An analysis of a subgroup (6814 participants) of the EuroSIDA study cohort verified that clinical outcome correlates strongly with most recent CD4+ T-cell or HIV RNA level, regardless of ART regimen used. In particular, it is worth noting that those with CD4 count ≤350 cells/mm^3 ^were at increased risk of AIDS or death than higher CD4 counts (rate ratio ≥3.39 vs ≤1.57), while the risk of these outcomes was substantially lower at HIV-RNA <500 copies/mL compared to >50 000 copies/mL (rate ratio 0.22 vs 0.61) [64]. These findings support the continued use of HIV-RNA and CD4 count as markers of disease progression on any HAART regimen [6,51,65].

There is no doubt that monitoring viral load is critical to assessing the efficacy of ART [65-68]. Findings from multiple studies reinforce the association between greater virological suppression and sustained virological response to ART [6,50]. Guidelines define virological failure as either a failure to achieve an undetectable HIV-RNA (<50 copies/mL) after 6 months or a sustained HIV-RNA >50 copies/mL or >400 copies/mL following suppression below this level [6]. A greater than threefold increase in viral load has been associated with increased risk of clinical deterioration and so this value is recommended to guide therapeutic regimen change in the developed world [69,70]. Several studies have shown a significant correlation between HIV-RNA >10 000 copies/mL and increased mortality and morbidity, and therapeutic switching should occur prior to this point [49]. The World Health Organisation recommends that this level be considered the definition of virological failure in resource limited settings [49].

Some patients experience intermittent episodes of low-level viremia followed by re-suppression below detectable levels, known as "blips". A very detailed study by Nettles et al. [71] defined a typical blip as lasting about 2.5 days, of low magnitude (79 copies/ml) and requiring no change in therapy to return to <50 copies/ml. Havlir et al. [72] and Martinez et al. [73] demonstrated that there was no association between intermittent low-level viremia and virological failure. Intermittent viremia does not appear to be a significant risk factor for disease progression. Nevertheless, it must be distinguished from true virological failure, which is consistently elevated HIV-RNA, as defined above. Continued follow up is essential.

Even in patients achieving apparently undetectable HIV-RNA levels, the HIV virus persists through the infection of memory T-cells [74,75]. This 'viral reservoir' has an extremely long half life and remains remarkably stable even in prolonged virological suppression [74,76,77]. Responsible for the failure of ART to eradicate infection, regardless of therapy efficacy, it may also contribute to the 'blips' described above [76]. Like intermittent viremia, its effect on disease progression appears trivial, being mainly of therapeutic importance [78,79]. However, as the long-term clinical outcomes of viral resistance and sub-detection viral replication become clearer, its significance may increase [15].

### Resistance mutations

Drug resistance is a strong predictor of virological failure after HAART, with a clear relationship seen between the number of mutations and virological outcome [56,80-84]. Hence maximal suppression of viral replication, with the parallel effect of preventing the development of resistance, is essential to optimise both response to treatment and improvement in disease progression [85,86].

The transmission of resistant virus is a serious reality, with implications for the efficacy of initial regimens in ART naïve patients. Prevalence of resistance mutations amongst seroconverters varies according to geographic location, with inter-country prevalence varying from 3–26%. This reinforces the need to gain local data, especially as resistance increases with increasing HAART use [87]. In the USA, the prevalence of primary (transmitted) resistance was 24.1% in 2003–2004, an almost two-fold increase from the 13.2% prevalence recorded in 1995–1998 [88]. European prevalence for 2001–2002 was 10.4%, which, although lower than the US figures, remains quite high [86,87]. Pre-treatment resistance testing has been shown to reduce the risk of virological failure in patients with primary drug resistance [6,50]. The DHHS guidelines suggest that pre-treatment resistance testing in ART naïve patients may be considered if the risk of resistance is high (ie: population prevalence ≥5%) while the British HIV Association (BHIVA) recommends testing for transmitted resistance in all newly diagnosed patients and prior to initiating ART in chronically infected patients [49]. In resource-limited settings, resistance testing may not be readily available; however, in such locations, primary resistance is likely to be rare, and need for pre-treatment resistance testing is lower [89]. An exception to this rule is the case of child-bearing women who have received intrapartum nevirapine, in whom poorer virological responses to post-partum nevirapine based regimens have been seen (49% vs 68% achieved HIV-RNA <50 copies/mL at 6 months) [49,89]. Regardless of the setting, there is a need for surveillance of local drug resistance prevalence [1,2,90].

### Chemokine receptor tropism

CCR5 and CXCR4 chemokine receptors act as co-receptors for HIV virions. Proportionately greater tropism for one or the other of these receptors has been associated with different rates of disease progression. Slowly progressing phases of infection are associated with predominance of the "R5 virus strains" that ligate the CCR5 receptor, mainly present on activated immune cell surfaces (including macrophages). "X4 strains" showing tropism for CXCR4, expressed by naïve or resting T-cells, and dual-tropic R5X4 strains, increase proportionately in the later stages of disease and are associated with more rapid clinical and immunological deterioration [1,2,90]. 'X4' strains have been associated with greater immune activation, suggesting a possible mechanism for their effects on disease progression [27]. Patients with predominantly X4 strains have been found to have lower CD4 counts, but correlations with viral load have been inconsistent [90-92]. Some host genetic phenotypes namely CCR5-Δ32 and SDF-1'A, affect R5 strain binding and are associated with delayed disease progression [93-95].

It is evident that even under effective HAART suppression [96], the predominant viral strain can change from R5 to X4 [90,92,97]. Additionally, about 50% of triple therapy experienced patients have been found to harbour X4 strains, a far greater proportion than the 18.2% seen in an ART naïve population [98]. The evidence for a difference in survival between those on HAART with X4 strains and those with R5 strains is difficult to interpret. Brumme et al. [98] suggested that a group of patients with the 11/25 envelope sequence (a highly specific predictor of the presence of X4 strains) had higher mortality and poorer immunological response to HAART despite similar virological responses to those without the 11/25 sequence. In contrast, a later study indicated that after adjustment for baseline characteristics, X4 strains were not associated with a difference in survival or response to HAART [99]. As can be seen, the effects of chemokine receptor tropism remain controversial and as yet, there is no clear evidence that monitoring or measuring these parameters will be useful clinically.

### Viral subtype and race

Complicating the assessment of the effect of viral subtype on disease progression are the potential confounders such as race, prevalence of various opportunistic infections and access to health care. Subtype C affects 50% of people with HIV and is seen mostly in Southern and Eastern Africa, India and China. Subtype D is found in East Africa and Subtype CRF_01 AE is seen mainly in Thailand. Caucasians are predominantly infected by subtype B, seen in 12% of the global HIV infected population [99]. The majority of research on all aspects of HIV has been performed amongst subtype B-affected individuals. The implications for treatment practice are obvious should differences in viral pathogenicity or disease progression exist between subtypes [99,100].

Rangsin et al. [100] noted median survival times in young Thai men (Type E 97%) of only 7.4 years, significantly shorter than the 11.0 years reported by the mainly Caucasian CASCADE cohort (Type B ≥50%). Hu et al. [101] found differences in early viral load between those with Type E (n = 103) when compared to Type B (n = 27) in Thai injecting drug users. Kaleebu et al. [102] studied a large cohort in Uganda, providing the strongest evidence for a difference in survival between A and D subtypes. However, analysis of a small cohort in Sweden reported no difference in survival rates between subtypes A-D [103]. Only Rangsin et al. [100] and Hu et al. [101] studied cohorts with estimable seroconversion dates.

It is difficult to control for the multiple potential confounding factors in research measuring the influence of subtype on disease progression. Geretti [99] remarked that the evidence for survival and disease progression rate differences between subtypes is currently inadequate to draw any definitive conclusions. Ongoing research is essential not only to determine the effect of subtype on disease progression but also to evaluate response to therapy.

Many of the confounding factors affecting subtype investigation also confound research into the effect of race on disease progression. Studies with clinical endpoints have found no significant relationship between race and disease progression [104,105], while another study of clinical response to HAART suggested disease progression appears to correlate more strongly with other factors (eg: depression, drug toxicity) than with race per se [106]. In support of this, data from the TAHOD database^ii ^(see Appendix 1 for details) suggests that responses to HAART among Asians are comparable to those seen in other races [11]. Evidence for racial variation in viral loads and CD4 counts has not been consistent and confounders have been difficult to exclude [107-109]. Morgan et al. [110] reported a median survival time of 9.8 years amongst HIV infected Ugandans which does not differ greatly from the 11.0 years reported by the mainly Caucasian CASCADE cohort. Race as an independent factor does not appear to play a part in the rate of disease progression independently of confounders such as psychosocial factors, access to care and genetically driven response to therapy.

### Host genetics

An understanding of the effect of host genetics on disease susceptibility and progression has significant implications for the development of therapies and vaccines [95]. Host genetics impact HIV infection at two main points: (i) cell-virion fusion, mediated primarily by the chemokine receptors CXCR4 and CCR5 and their natural ligands, and (ii) the host immune response, mediated by Human Leukocyte Antigen (HLA) molecules [95,111].

Polymorphisms of the genes controlling these two pathways have been extensively studied and multiple genetic alleles that have been found to correlate with either delayed or accelerated disease progression [95,111,112].

HLA molecules provide the mechanism by which the immune system generates a specific response to a pathogen. As has been described earlier, the diversity of HIV-specific immune responses plays a crucial role in containment of the virus and it is HLA molecules that control that diversity. Thus, HLA polymorphisms should affect disease progression. Investigation of the effect of specific alleles has found that heterozygosity of any MHC Class I HLA alleles appears to delay progression, while rapid progression has been associated with some alleles in particular, for example, HLA-B35 and Cω4[95]. The HLA-B57 allele, present in 11% of the US population and around 10% of HIV-positive individuals, has been linked to long-term non-progression, a lower viral set-point and fewer symptoms of primary HIV infection [95,112].

In addition to the effect of genetic polymorphisms on the natural history of infection, host genetic profile can influence the response to HAART [113]. In Australia, the presence of the HLA-B5701 allele accounts for nearly 90% of patients with abacavir hypersensitivity. Drug clearance also varies significantly between racial groups due to genetic variations in CYP enzyme isoforms [114]. For example, polymorphisms of CYP2B6 occurring more frequently in people of African origin are associated with three-fold greater plasma efavirenz concentrations, leading to a greater incidence of central nervous system toxicity amongst this group [115]. Potential outcomes of such phenomena include treatment discontinuation in the case of toxicity or hypersensitivity and drug resistance when medications are ceased simultaneously causing monotherapy of the drug with the prolonged half life [114]. Genetic screening in order to guide choice of therapy is already underway in Australia for HLA-B57 alleles related to abacavir hypersensitivity [114]. Studies of host genetics appear likely to significantly influence the clinical management of HIV in the future.

## Other host factors

Studies of many of these factors usually assume equality of access to care for members of the study population. A survey of people living with AIDS in New York city found that female gender, older age, non-Caucasian race and transmission via injecting drug use or heterosexual intercourse were all associated with significantly higher mortality. This most likely reflects the poorer access to health care and other sociological disparities experienced by these groups [116].

### Age

Age at seroconversion has repeatedly been found to have considerable impact on the future progression of disease. Concurring with earlier studies, the CASCADE collaboration [62] found a considerable age effect correlating with CD4 count and HIV-RNA, across all exposure categories, CD4 count and HIV-RNA strata in an analysis of multiple international seroconversion cohorts, reinforcing these findings again recently [10,117,118]. Table 1 clearly demonstrates the importance of stratification by age, CD4 count and HIV-RNA as predictive of the short term risk of AIDS. There is a clear relationship between increasing risk with increasing age. For example, a 25 year old with a CD4 count of 200 and HIV-RNA level of 3000 has one third the risk of disease progression when compared to a 55 year old. This raises the issue of whether or not older patients should be treated at higher CD4+ T-cell counts [10].

Older age is associated with lower CD4 counts at similar time from seroconversion which may explain the relationship between age and disease progression [57,119]. However, age disparities seem to diminish with HAART treatment; CD4 counts and HIV-RNA levels becoming more useful prognostic indicators [119]. It appears that the age effect seen on HAART treatment is closer to the natural effect of aging rather than the pre-treatment, HIV-related increase in mortality, suggesting that HAART attenuates the effect of age at seroconversion on HIV disease progression [120].

### Gender

Mean HIV-RNA has been found to vary between men and women for given CD4 count strata [107,121-123]. Low levels of CD4+ T-cells (<50 cells/mm^3^) are associated with higher mean HIV-RNA in women (of the order of 1.3 log_10_copies/mL) than in men within the same CD4 count stratum. Conversely, at higher CD4+ T-cell levels (>350 cells/mm^3^), mean HIV-RNA has been noted to be 0.2–0.5 log_10_copies/mL lower in women [124]. Despite HIV-RNA variation, disease progression has not been seen to differ between the genders for given CD4 counts [121,124,125]. On this basis, the current DHHS *Guidelines for the use of Antiretroviral Drugs in HIV-1 Infected Adults and Adolescents *state that there is no need for sex-specific treatment guidelines for the initiation of treatment given that antiretroviral therapy initiation is guided primarily by CD4 count [6].

### Mode of transmission

Comparing disease progression rates between transmission risk groups has led to conflicting findings. An early study found significantly faster progression amongst homosexuals than heterosexuals [126]. However, more recent studies analysing much larger cohorts reported no difference in disease progression rates following adjustment for age and exclusion of Kaposi's sarcoma as an AIDS defining illness [62,127,128]. Prins et al. [127] noted that injecting drug users have a very high other-cause mortality rate that could confound results failing to take this into account.

The CASCADE collaboration [120] examined the change in morbidity and mortality between the pre- and post-HAART periods. They found a reduction in mortality in the post-HAART era amongst homosexual and heterosexual risk groups but no such change in injecting drug users. This apparently higher risk of death than other groups may be related to poor therapy adherence, less access to HAART and the higher rate of co-morbid illnesses such as Hepatitis C. Other factors may have a larger role to play in clinical deterioration than the mode of transmission.

### Psychosocial factors

Understanding the interaction between physical and psychosocial factors in disease progression is important to maximise holistic care for the patient. Several studies have found significant relationships between poorer clinical outcome and lack of satisfaction with social support, stressful life events, depression and denial-based coping strategies [129-132]. Other studies have found strong correlations between poorer adherence to therapy and depression, singleness and homelessness [106,133]. Patient management should include consideration of the psychosocial context and aim to provide assistance in problem areas.

## Resource limited settings

The three elements of host, immunological and virological factors obviously synergise to influence the progression of HIV infection, however, a few additional factors may hold prognostic value. While CD4 count and HIV-RNA are the gold standard markers for disease monitoring, when measurement of these parameters is not possible surrogate markers become important. Markers investigated for their utility as simple markers for disease progression in resource-limited settings include delayed type hypersensitivity responses (DTH), total lymphocyte count (TLC), haemoglobin and body mass index (BMI).

### Delayed type hypersensitivity

Mediated by CD4+ T-lymphocytes, DTH-type responses give an indication of CD4+ T-cell function in vivo. It has been shown that DTH responses decline in parallel with CD4+ T-cells resulting in a corresponding increase in mortality [134,135]. Failure to respond to a given number of antigens has been suggested as a marker for the initiation of ART in resource-limited settings [135,136].

Improved DTH responses have been noted with ART, although the degree of improvement appears dependent on the CD4+ nadir prior to HAART initiation [137-139]. This holds implications for the timing of initiation of treatment, as delayed treatment and hence low nadir CD4 counts may cause long-term immune deficits [139]. There is a need for further research in resource-limited settings to determine the utility of DTH testing as both a marker for HAART initiation and a means of monitoring its efficacy.

### Total lymphocyte count

Another marker available in resource-limited countries, total lymphocyte count (TLC), has been investigated as an alternative to CD4+ T-cell count. Current WHO guidelines recommend using 1200 cells/mm^3 ^or below as a substitute marker for ART initiation in symptomatic patients [140]. Evidence for the predictive worth of this TLC level is encouraging, with several large studies confirming the significant association between a TLC of <1200 cells/mm^3 ^and subsequent disease progression or mortality [135,141,142]. Others propose that rate of TLC decline should be used in disease monitoring as a rapid decline (33% per year) precedes the onset of AIDS by 1–2 years [142,143]. Disappointingly, there is generally a poor correlation between TLC and CD4 count at specific given values.

While TLC measurement has been validated as a means of monitoring disease progression in ART-naïve patients, its use for therapeutic monitoring is questionable and not recommended [49,144-146].

### Body mass index

The body mass index (BMI) is a simple and commonly used measure of nutritional status. Its relationship to survival in HIV infection is important for two main reasons. Firstly, 'wasting syndrome' (>10% involuntary weight loss in conjunction with chronic diarrhoea and weakness, +/- fever) is considered an AIDS defining illness according to the CDC classification of disease [1]. Secondly, the ease of measurement of this parameter makes it potentially highly useful as a marker for the initiation of ART in resource limited countries.

Like TLC, long-term monitoring of BMI is predictive of disease progression. A rapid decline has been noted in the 6 months preceding AIDS although the sensitivity of this measure was only 33% [145,146]. A baseline BMI of <20.3 kg/m^2 ^for men and <18.5 kg/m^2 ^for women is predictive of increased mortality, even in racially diverse cohorts, with a BMI of 17–18 kg/m^2 ^and <16 kg/m^2 ^being associated with a 2-fold and 5-fold risk of AIDS respectively [147-149].

In combination with other simple markers such as haemoglobin, clinical staging and TLC, a BMI <18.5 kg/m^2 ^shows similar utility to CD4 count and HIV-RNA based guidelines for the initiation of HAART [150,151]. A sustained BMI <17 kg/m^2 ^6 months after HAART initiation has been associated with a two-fold increase in risk of death [152].

As can be seen, measurement of the body mass index is a simple and useful predictor of disease progression. A BMI of <18.5 kg/m^2 ^was consistently strongly associated with increased risk of disease progression and may prove to be a valuable indicator of the need for HAART.

### Haemoglobin

Haemoglobin levels reflect rapidity of disease progression rates and independently predict prognosis across demographically diverse cohorts [151,153]. Rates of haemoglobin decrease also correlate with falling CD4 counts [135,141].

There have been suggestions that increases in haemoglobin are predictive of treatment success when combined with a TLC increase [143]. While racial variation in normal haemoglobin ranges and the side effects of antiretroviral agents such as zidovudine on the HIV infected bone marrow must be taken into account [144], monitoring haemoglobin levels shows utility in predicting disease progression both before and following HAART initiation.

## Conclusion

The evolution of HIV infection from the fusion of the first virion with a CD4+ T-cell to AIDS and death is influenced by a multitude of interacting factors. However, in gaining an understanding of the prognostic significance of just a few of these elements it may be possible to improve the management and long-term outcome for individuals. Host factors, although unalterable, remain important in considering the prognosis of the patient and guiding therapeutic regimens. Furthermore, research into host-virus interactions has great potential to enhance the development of new therapeutic strategies.

Immunological parameters such as levels of CD38 expression and the diversity of HIV-specific cytotoxic lymphocyte responses allow insight into the levels of autologous control of the virus. Virological monitoring, including drug resistance surveillance, will continue to play a considerable role in the management of HIV infection. Additionally, as access to antiretroviral therapy improves around the world, the utility of, and need for, low-cost readily available markers of disease is evident. As with any illness of such magnitude, it is clear that a multitude of factors must be taken into account in order to ensure optimum quality of life and treatment results.

## Competing interests

The author(s) declare that they have no competing interests.

## Appendix 1

^i ^The "Concerted Action of Seroconversion to AIDS and Death in Europe" (CASCADE) collaboration includes cohorts in France, Germany, Italy, Spain, Greece, Netherlands, Denmark, Norway, UK, Switzerland, Australia and Canada

^ii ^The "TREAT Asia HIV Observational Database" (TAHOD) database contains observational information collected from 11 sites in the Asia-Pacific region, encompassing groups from Australia, India, the Philippines, Malaysia, China, Singapore and Thailand.
